# Callous-Unemotional Traits and Academic Performance in Secondary School Students: Examining the Moderating Effect of Gender

**DOI:** 10.1007/s10802-019-00545-2

**Published:** 2019-04-16

**Authors:** Elisabeth Bird, Celine Y. Chhoa, Emily Midouhas, Jennifer L. Allen

**Affiliations:** Department of Psychology and Human Development, UCL Institute of Education, University College London, 20 Bedford Way, London, WC1H 0AL UK

**Keywords:** Academic achievement, Callous-unemotional traits, Gender differences, Psychopathic traits, School grades, Youth psychopathy

## Abstract

Callous-unemotional (CU) traits and male gender are both known risk factors for poor academic outcomes in children and adolescents. However, despite gender differences in CU trait severity, comorbid difficulties and correlates of CU traits, research has yet to examine whether the CU traits and male gender may work together to increase risk for poor academic performance. That is, whether boys high in CU traits perform more poorly across academic disciplines than girls high in these traits. This study therefore aimed to investigate i) the relationships between CU traits, student gender and English, Science and Math grades, and ii) whether gender moderates the association between CU traits and academic outcomes. Participants were 437 children aged 11 to 14 years (mean age 12.50 years; 49% girls; 85% White) attending a state secondary school in England. Students reported on CU traits and externalizing problems and their English, Math and Science grades were gathered from school records. Using hierarchical linear modelling, CU traits were found to be significantly related to lower English, Math and Science grades when controlling for age, gender, sociodemographic disadvantage and externalizing problems. CU traits were significantly related to lower Science grades for boys but not girls. However, gender did not moderate the association between CU traits for English or Math grades. Findings enhance our understanding of how child characteristics may interact to increase the likelihood of poor school outcomes, and therefore help us to identify youth at-risk for poor academic performance.

The last two decades have seen a surge of interest in investigating psychopathic features in youth as one way of explaining the heterogeneous nature of risk factors and outcomes for antisocial behaviour. Three temperament dimensions in children corresponding to the multifaceted model of psychopathic traits in adults have been identified, encompassing callous-unemotional (CU) traits (affective dimension), narcissism (interpersonal dimension) and impulsivity (behavioural dimension). CU traits comprising low empathy, lack of guilt, restricted affect and low concern for performance are considered to be the core feature of psychopathy in children (Frick et al. 2014). These traits are related to early-onset conduct problems and more varied, severe and chronic antisocial behaviour (Obradović et al. 2007; Salekin 2008). CU traits are associated with relatively distinct neurological and biological correlates including reduced recognition and responsiveness to others’ distress cues (Blair et al. 2005; Ciucci et al. 2015, 2018). They appear motivated by social dominance and forced respect, reporting low interest in social approval or forming positive relationships with others (Pardini 2011). Children high in CU traits have more positive expectations about the outcomes of aggression and less concern for its consequences including anticipated feelings of remorse, disciplinary action, or victim distress (Pardini and Byrd 2012). The presence of these unique correlates and poor prognostic indicators have led to the inclusion of CU traits as a specifier for Conduct Disorder in the fifth edition of the Diagnostic and Statistical Manual for Mental Disorders (American Psychiatric Association, hereafter APA, 2013), under the term limited prosocial emotions (LPE).

The majority of research investigating CU traits in social contexts has focussed on the family environment. The few studies conducted in the school setting indicate youth with CU traits display more aggressive, deceitful and manipulative behaviour in the classroom than their low CU peers (Allen et al. 2016; Waschbusch et al. 2015), greater conflict and less closeness with teachers and reduced responsiveness to teacher classroom management strategies (Allen et al. 2016; Crum et al. 2016; Horan et al. 2016). They are more likely to bully peers both directly and indirectly (Ciucci and Baroncelli 2014; Muñoz et al. 2011), and to report more impaired peer relationships, low levels of peer support and school connectedness (Fanti et al. 2017; Haas et al. 2018). Few studies have examined the link between CU traits and academic performance, despite the inclusion of lack of concern about school performance as a symptom of the LPE specifier. Furthermore, items in the Inventory of Callous Unemotional traits (ICU; Frick 2004) focus on attitudes that are likely to promote poor academic outcomes (e.g., ‘I care about how well I do at school or work’). Investigating the relationship between CU traits and academic outcomes may assist in identifying children at risk for poor academic performance and help schools to direct resources towards students most in need of support.

There is a well-established link between low verbal IQ and antisocial behaviour in youth (Allen 2017), with this association present even when accounting for minority ethnicity status, social disadvantage and test-taking motivation (Lynam et al. 1993). However, children with elevated CU traits possess a similar verbal IQ to their low CU peers (Allen et al. 2013; Loney et al. 1998), suggesting that there may be heterogeneous risk pathways for poor academic outcomes in antisocial youth with high versus low levels of CU traits. Several alternative explanations have therefore been put forward to explain poor academic performance in youth with elevated CU traits. The first proposes that youth high in CU traits perform poorly at school because their callous-unemotional interpersonal style extends to a lack of concern and indifference about their performance, leading to poor engagement with academic work (DeLisi et al. 2011). DeLisi et al. argued further that the insensitivity of children high in CU traits to punishment and social disapproval (e.g., teacher discipline, peer rejection), prevents youth from learning to modify their disruptive behaviour and engage in school work. In a similar vein, Horan et al. (2016) speculated that CU traits may elicit harsh responses from teachers, as well as less teacher encouragement, questioning and feedback, with reciprocal negative teacher-child interactions and a conflictual teacher-student relationship exacerbating poor adjustment at school. A final explanation suggests that emotion processing deficits in individuals with psychopathic traits may contribute to reading comprehension difficulties. Hiatt and Newman (2006) argued that while the language ability of individuals with psychopathic traits is broadly intact, they may have difficulty with more subtle or contextual aspects of language, particularly when comprehension is reliant on an understanding of emotional connotations.

Gender differences have also been documented relating to the severity, correlates and comorbid difficulties seen in youth with CU traits. For example, higher levels of CU traits, antisocial behaviour and impulsivity are evident for boys compared to girls (Cardinale and Marsh 2017; Essau et al. 2006; Fanti 2013). CU traits in girls are associated with more severe internalizing problems and higher levels of affective empathy compared to boys (Cardinale and Marsh 2017; Stickle et al. 2012). In addition, there is a well-documented female advantage for teacher-assigned grades (effect size *d* = 0.23), with a recent meta-analysis by Voyer and Voyer (2014) showing that a small but significant female advantage was consistent over time, across disciplines and levels of schooling. The female advantage was most prominent during middle high school, and in the discipline of English/Languages (*d* = 0.45), followed by Science (*d* = 0.23) and Math (*d* = 0.23). A number of explanations have been put forward for this female advantage ranging from gender differences in temperament (lower activity/impulsivity and higher effortful control in females), to sex-biased treatment and expectations (greater teacher and parental encouragement of girls), a female tendency to possess a mastery versus performance orientation to learning, or to a combination of these factors (Dweck 1986; Else-Quest et al. 2006; Hartley and Sutton 2013; Kenney-Benson et al. 2006). The greater severity of CU traits and comorbid problems in boys may therefore increase their risk for poor academic outcomes relative to girls, but this possibility remains to be tested.

Research has consistently found a significant relationship between CU traits in youth and poor achievement in school. DeLisi et al. (2011) assessed the reading comprehension of 432 predominantly Hispanic and African-American students (57% boys) in Years 7 and 8 in schools located in socially disadvantaged areas. Most students were ‘struggling readers’ (*n* = 354), with the remainder comprising typically developing students (*n* = 78). Latent class analyses revealed that the two groups with the highest levels of CU traits had lower scores on reading comprehension. These groups comprised predominantly male students (72% and 78%, respectively) and students with symptoms of inattention, hyperactivity, and impulsivity. The remaining two groups with the lowest ICU total scores did not differ from the former two groups in IQ scores or ethnicity. Vaughn et al. (2011) examined the relationships between CU traits, externalizing symptoms and reading achievement in the same sample using multiple regression models. Pupils high in CU traits scored lower on reading comprehension and achievement, controlling for IQ and symptoms of inattention, hyperactivity and impulsivity.

A more recent study examined the association between CU traits and performance in standardized Math and Reading exams in 3rd grade students (*N* = 942, 51% girls) who were predominantly Hispanic or Black/African-American (Horan et al. 2016). Items assessing CU traits were selected from i) a child-report measure of empathy and ii) teacher-report measures of aggression, social competence, student responsibility and teaching stress based on their content similarity to items from the ICU (Frick 2004) and the Childhood Psychopathy Scale (Lynam 1997). Factor analysis indicated that the resultant measure of CU traits consisted of three scales: Callousness, Uncaring and Empathy. These scales were used to create two distinct profiles of CU traits: children high in Callousness and Uncaring and low in Empathy (*n* = 130) and children high in Callousness, Uncaring and Empathy (*n* = 120), with the remainder of the sample forming a low CU reference group. Callousness and Uncaring were based on teacher report measures and Empathy on child report. Membership of either high CU traits group was significantly related to poor performance in Math and Reading exams. Male gender was related to poorer achievement in Reading, but not Math. A potential interaction between gender and CU traits was not examined.

Studies have also found links with CU traits and poor academic outcomes using teacher questionnaire ratings of academic impairment. Ciucci et al. (2014) examined relationships between teacher-report of CU traits and academic achievement in 540 Italian children aged 10 to 16 years (52% girls). ICU total scores were related to lower achievement, assessed using mean ratings of teacher evaluations of student performance collapsed across all academic subjects. Fanti et al. (2017) also examined the links between CU traits and teacher ratings of academic impairment. They identified four trajectory groups on the basis of parent ICU report in a large sample of 9 year old Cypriot children (*N* = 1200) across 3 time points within a one-year period: stable high, increasing, decreasing and low. The gender-by-group interaction was not significant. Children with high-increasing CU traits over a 1 year period showed the poorest academic performance, followed by children in the low-increasing group; while the low and decreasing groups showed similar and higher levels of academic performance to the other two groups. Boys were higher in CU traits, impulsivity and social competence than girls and were lower in empathy and school connectedness. Once again a potential interaction between gender and CU traits was not examined.

## The Present Study

Evidence indicates that CU traits are independently associated with poor academic outcomes even when controlling for externalizing problems (Horan et al. 2016; Vaughn et al. 2011). This finding is consistent across primary and secondary school periods, using different assessment methods (teacher ratings, achievement tests) and occurred regardless of whether person-centred or variable-based approaches to analysis were employed. The two studies that examined performance beyond reading comprehension/achievement also support a link between CU traits and poor academic performance (Ciucci et al. 2014; Horan et al. 2016). Boys with elevated CU traits may be at increased risk for lower grades due to an increased severity of CU traits and externalizing problems along with lower levels of empathy and social competence (Cardinale and Marsh 2017; Fanti 2013; Fanti et al. 2017). However, while two past studies examined direct links between CU traits, gender and academic performance (Ciucci et al. 2014; Fanti et al. 2017), the joint role of CU traits and child gender on academic performance was not examined. Furthermore, these studies both used teacher ratings of academic performance which are susceptible to bias.

This study aims to investigate the relationship between CU traits, gender and school grades in pupils attending a state secondary school in England. Following the transition to high school, academic work becomes more varied and challenging with students simultaneously expected to show greater independence in their studies. Risk for poor outcomes may therefore be heightened during this period, as students can feel negative about their academic potential, put in less effort and give up more quickly (Midgley and Urdan 1992). Furthermore, the female advantage in teacher-assigned grades reaches its peak during middle high school relative to elementary and senior high school (Voyer and Voyer 2014). A better understanding of how CU traits and gender interact to influence school success may help to identify students at-risk for poor outcomes and enable more targeted intervention. We used the total ICU score of the child-report version of the ICU (Frick 2004), given support for CU traits as a general factor and concerns about the use of the ICU subscales (Ray and Frick 2018). We controlled for externalizing behaviors given longstanding evidence for a relationship between antisocial behaviour, symptoms of inattention and hyperactivity/impulsivity and both cognitive and academic impairment (Allen 2017; Arnold 1997; Hinshaw 1992).

Student grades were based on performances on curriculum set assessments across three major academic disciplines: English, Science and Math. We investigated performance in these disciplines separately given that the female academic advantage is more apparent for English than other disciplines (Voyer and Voyer 2014), and differences in teaching methods across English, Maths and Science in the UK (Baines et al. 2003). The current sample overlaps with a previous mixed methods study that examined CU traits, teacher-child interaction and academic motivation from teachers’ perspectives (Allen et al. 2018). Findings indicated that teachers found it difficult to form good relationships with students high in CU traits, with the quality of the teacher-student relationship related to the frequency and severity of disruptive behaviour in class, responsiveness to teacher discipline and reward-based strategies, and academic motivation. Furthermore, teachers viewed students with elevated CU traits as low in academic motivation despite possessing the ability to do well. The present study builds on this work by examining relationships between CU traits, gender and actual student grades. We expected that CU traits would significantly predict lower Math, English and Science grades. A second prediction was that gender would moderate the association between CU traits and academic performance, such that the combination of male gender and higher CU traits would significantly predict lower grades. Conversely, female gender is predicted to protect against the negative impact of CU traits, such that girls with higher CU traits would outperform boys higher in CU traits across all subjects.

## Method

### Participants

Participants comprised 437 children in Years 7 to 9 attending a state secondary school in a rural town in the east of England (population ~42,800). Of the 503 children invited to participate in the present study, 66 declined to complete the questionnaires, giving a participation rate of 87%. Child participants included 216 girls and 221 boys aged 11 to 14 years (*M* = 12.50 years, *SD* = 0.96). The majority of children were White (85%) and had English as their first language (77%). The remainder of the sample (*n* = 17) identified their ethnicity as follows: Black, Mixed Black and White, Asian, or Mixed White and Asian. Most children reported living with their original two-parent family (60%), followed by a step/blended family (21%), with the remainder living in a single parent household (16%), or with an extended family member (3%). Eleven percent of children were eligible for free school meals (*n* = 46). Children in England meet eligibility criteria for free school meals if their parent(s) or guardian(s) are on a low income or out of work. Free school meal eligibility is reliably used as an indicator of socioeconomic disadvantage/a proxy of socioeconomic status in academic research on educational outcomes in England (Taylor 2017). Table 1 shows participant sociodemographic characteristics relative to the mean values for England. Classroom size ranged from 15 to 31 children (*M* = 21.38, *SD* = 4.07), and the number of participating children per classroom ranged between 12 and 30 (Mdn = 21.00). Students were in different classes for English, Science and Math depending on their ability level, and remained in the same class for each discipline during the entire school year.

**Table 1:** Participant sociodemographic characteristics

	Sample (*N* = 437)	Mean values for England (%)
Male	221 (51%)	49^a^
Single-parent family (*N* = 434)	68 (16%)	22^b^
Receives free school meal (*N* = 427)	46 (11%)	12.9^c^
English as an additional language (*N* = 431)	99 (23%)	16.2^c^
Non-white ethnicity (*N* = 437)	64 (15%)	14^a^

^a^Data from 2017a Census, Office for National Statistics, London

^b^Data from 2017b Labour Force Survey, Office for National Statistics, London

^c^Data from 2017 School Census, Department for Education, London

#### Teacher Participants

Teacher participants consisted of eight women and four men aged 23 to 51 years (*M* = 35.27, *SD* = 10.43). Teachers taught English (*n* = 5), Maths (*n* = 2), or Science (*n* = 5). Teachers completed the Strengths and Difficulties Questionnaire (SDQ; Goodman 1997) for a randomly selected subset of students who i) scored in the top 25% of the student-report total ICU score (high CU group; *n* = 24) and ii) who scored below the median on the total ICU score (low CU group; *n* = 23). Teachers declined to complete questionnaires for 9 students, leaving a final subsample of 38 students (high CU group *n* = 11; low CU group *n* = 27). The aim of this smaller selected sample was to obtain more information about students without placing a burden on teachers.

### Procedure

Following the receipt of approval from the University College London Institute of Education ethics board, permission to approach students and teachers to participate in the study was sought from the school. Information and consent forms and reply slips were then mailed to parents of all pupils. This study used ‘opt-out’ consent, with parents given a week to return the reply forms if they did not consent for their child to take part. No reply slips were returned. Parent opt-out consent helps to avoid low response rates and biased samples, leading to incomplete and potentially misleading findings. An opt-in sample is likely to be skewed towards students with lower levels of externalizing problems, social disadvantage and higher levels of academic achievement, which would be problematic given that our research aims to examine academic outcomes in relation to CU traits. The study took place during regular lesson time in class groups. The investigator informed pupils that the study focussed on the relationship between academic attainment and the behaviours and attitudes of young people towards parents, teachers and peers. Students were informed that their responses were confidential, and that they could leave the questionnaires uncompleted or omit items without giving a reason. Students completed the questionnaires individually under exam conditions and were instructed to raise their hand if they did not understand any of the items so that the investigator could provide assistance. On completion of questionnaires, students were given the opportunity to ask any questions and thanked for their participation. Teachers completed questionnaires about children in the selected sample following the receipt of their written informed consent.

### Measures

#### Callous-Unemotional Traits

The Inventory of Callous Unemotional Traits (ICU; Frick 2004) was used to assess student report of callous-unemotional (CU) traits. The ICU is a 24-item scale used to measure three dimensions of CU traits: Unemotional (e.g., “I hide my feelings from others”, 5 items), Uncaring (e.g., “I care about how well I do at school or work” - reversed-scored, 8 items), and Callousness (e.g., “I do not care who I hurt to get what I want”, 11 items). Items for each scale are rated on a 4-point Likert scale from 0 (not true at all) to 3 (definitely true). The ICU total score is calculated by summing all subscale items and has shown good validity and reliability in adolescent samples (Essau et al. 2006; Kimonis et al. 2008). Consistent with past research (Kimonis et al. 2008; Ray et al. 2016), items 2 and 10 had item-total correlations (ITCs) below 0.10, and following their removal, Cronbach’s alphas increased from 0.77 to 0.79. Thus we used the ICU total scale with those two items removed in all analyses.

#### Externalizing Problems

Items to assess student report of externalizing problems were selected from the Reward Sensitivity of the child version of the revised Sensitivity to Punishment and Sensitivity to Reward Questionnaire (SPSRQ-C; Colder et al. 2011). The 18-item Reward Sensitivity scale consists of three subscales: Impulsivity (7 items), Reward Responsivity (7 items) and Competitive Drive (4 items). Items are rated on a 5-point Likert scale from 1 (strongly disagree) to 5 (strongly agree). We did not use the standard self-report SPSRQ as its items were not appropriate for the age of our sample. For example, it features items inappropriate for children (e.g., ‘Do you often take the opportunity to pick up people you find attractive?’; ‘When you start to play a slot machine, is it difficult for you to stop?’). Items in the parent-report SPSRQ-C were re-worded to be appropriate for child report. For example, ‘The possibility of obtaining social status moves your child to action, even if this involves not playing fair’ was re-worded to ‘The possibility of obtaining social status moves you to action even if this involves not playing fair’. Adolescent report on a modified version of the adult self-report SPSRQ, featuring a similar format (i.e., items presented as statements) and item coverage to the child-report version of the SPSRQ-C has shown good reliability and construct validity (Vandeweghe et al. 2016).

Items were selected from the Reward Sensitivity scale if they assessed antisocial behaviour or symptoms of ADHD (e.g., hyperactivity, deficits in response inhibition, attentional focusing and persistence). Examples of selected items include: ‘You often have trouble resisting the temptation to do forbidden things’, ‘You have difficulty staying focused on your schoolwork in the presence of an attractive alternative’ and ‘You like displaying your physical abilities even though it may involve danger’. A confirmatory exploratory factor analysis was then conducted on the 10 items selected to determine if any underlying structures existed that are consistent with the construct of externalizing problems. Following the removal of one item with a factor loading less than 0.3, the analysis produced a one-factor solution accounting for 34% of the total variance. The eigenvalue for this component was 3.04, with item loadings ranging from 0.41 to 0.67. This factor consisted of nine items – six from the Impulsivity scale, two from the Reward Responsivity scale and one from the Competitive Drive scale. Cronbach’s alpha for this externalizing problems scale was 0.75. Student report of externalizing problems was significantly related to teacher SDQ report of externalizing problems (*r* = 0.32, *p* = 0.05) for a subset of the sample (*n* =38).

#### Academic Performance

In England, children attending state schools are educated in line with the National Curriculum, which outlines the subjects to be taught and standards children should reach at each of four ‘key stages’. State schools must offer English, Math and Science as core or compulsory subjects to pupils in Key Stage 3 (KS3), consisting of pupils in Years 7–9. During KS3, students are assessed by compulsory teacher set curriculum assessments in relation to the National Curriculum programmes of study (see www.gov.uk/national-curriculum/overview). That is, teachers set multiple assignments and tests, with aggregated scores converted to final grades. Grades are awarded on the National Curriculum scale which ranges from 1 to 9, with higher scores indicating a higher level of academic attainment. Students’ current academic grades for English, Math and Science were collected from school records.

#### Sociodemographic Characteristics

In addition to the above measures, adolescents reported their age in years, gender (1 = male, 0 = female), English as an additional language, single-parent family, and eligibility for free school meals were all coded 1 = yes, 0 = no.

### Data Analysis

We first carried out descriptive analyses, including parametric correlations among the key study variables. Then we fitted two multiple linear regression models for each grade domain - English, Math and Science – resulting in a total of six models. Model 1 regressed grades on ICU total scores, externalizing problems and all sociodemographic variables. Model 2 tested the interaction effects of CU traits by gender. Regression models were fitted using MLwiN 3.00 (Charlton et al. 2019). Listwise deletion was used in all models. Final model samples ranged 398–404.

We fitted two-level multilevel models (Snijders and Bosker 1999) given our data had a hierarchical structure (students were nested within classes) and we wanted to avoid the underestimation of standard errors due to this clustering (Goldstein 1995). Initially, we tested the null hypothesis that there were no class differences in scores using the Likelihood Ratio Test to compare a null 2-level model with a single-level model. There were significant class differences for English and Science but not Math (English: *x*^*2*^ (1) = 72.65, *p* < 0.001; Math: *x*^*2*^ (1) = 32.33, *p* > .05; Science: *x*^*2*^ (1) =1.04, *p* < 0.001) indicating that a multilevel model is preferable to a single-level model for these subject grades. In null models (not presented), the intra-class correlations (ICCs) were 0.12 (English), 0.00 (Math) and 0.04 (Science) indicating that the variation in grades attributed to classes ranged from 0% to 12%.

At Level 1, we had students and, at Level 2, we had classes. There were eight English classes with a range of 26–87 students, nine Math classes with a range of 21–65 students and nine Science classes with a range of 21–107 students. In year 7, English was taught by five teachers, Science by four teachers and Math by four teachers. In year 8, English was taught by four teachers, Science by five teachers and Math by seven teachers. In year 9, English was taught by five teachers, Science by seven teachers and Math by six teachers. Most teachers (*N* = 26) taught within their respective discipline across either two or all three school years. Specifying a random intercept for classroom allowed students’ grades to vary by classroom for each subject.

## Results

### Descriptive Analyses

Table 2 shows descriptive statistics for all variables in the sample. The average ICU score prior to the removal of items 2 and 10 in our sample (*M* = 23.86) was equivalent to or higher than those previously obtained in community samples of adolescents, where mean scores ranged from 19.11 to 24.05 (Docherty et al. 2017; Essau et al. 2006; Fanti et al. 2009; Feilhauer et al. 2012; Roose et al. 2010). Two-tailed Pearson’s correlations between main study variables are presented in Table 3. Grades for all three subjects were significantly negatively correlated with the ICU total score. Maths and Science were significantly negatively correlated with externalizing problems, but there was no correlation with English grades (*p* = 0.27), With respect to sociodemographic factors, living in a single-parent family and English as an additional language were significantly negatively correlated with grades in Math and Science, but there was no correlation with English grades (*p* = 0.22 and *p* = 0.30, respectively). Free school meal eligibility was significantly negatively correlated with Science grades only (English, *p* = 0.31; Math, *p* = 0.36). Male gender was significantly negatively correlated with English grades only (Math, *p* = 0.68; Science, *p* = 0.08).

**Table 2:** Descriptive statistics of study variables for the sample

Variable	*n*	*M*	*SD*	*Range*	*Skewness (SE)*	*Kurtosis (SE)*
Age (years)	437	12.50	0.96	11–14	0.01(0.12)	−0.93(0.23)
Externalising problems	436	0.00	1.00	−2.49–2.67	0.25(0.12)	−0.17(0.23)
CU traits	435	21.28	7.88	3–45	0.31(0.12)	−0.34(0.23)
English grade	414	3.65	1.37	1–7	−0.33(0.12)	−0.66(0.24)
Math grade	414	3.90	1.20	1–8	−0.79(0.12)	0.49(0.24)
Science grade	420	4.08	1.04	1–9	0.07(0.12)	1.68(0.24)

*CU* Callous-unemotional

**Table 3:** Correlations between main study variables

Variable	1	2	3	4	5	6	7	8	9
1 Age									
2. Male	−0.04								
3. Single-parent family	0.08	0.06							
4. Free school meal	−0.07	0.13**	0.14**						
5. English as an additional language	−0.07	0.01	0.09	−0.04					
6. Externalising problems	−0.06	0.14**	0.04	0.06	0.01				
7. CU traits	0.09	0.14**	0.07	0.12*	−0.01	0.21**			
8. English grade	−0.46**	−0.16**	−0.06	−0.05	−0.05	−0.06	−0.16**		
9. Math grade	−0.14**	−0.02	−0.15**	−0.05	−0.11*	−0.19**	−0.18**	0.53**	
10. Science grade	0.09	−0.09	−0.15**	−0.17**	−0.15**	−0.19**	−0.22**	0.41**	0.72**

*CU* Callous-unemotional

*p < 0.05. **p < 0.01

### Multilevel Models

Results of the models are presented in Table 4.

**Table 4:** Effect estimates of CU traits, externalizing problems, and sociodemographic variables predicting English, Math, and Science grades

Variable	English		Math		Science	
	Model 1	Model 2	Model 1	Model 2	Model 1	Model 2
	β (SE)	β (SE)	β (SE)	β (SE)	β (SE)	β (SE)
Constant	11.62*** (0.84)	11.62*** (0.86)	6.57*** (0.78)	6.42*** (0.79)	3.38*** (0.69)	3.11*** (0.69)
Age	−0.59*** (0.07)	−0.59*** (0.07)	−0.17** (0.06)	−0.17** (0.06)	0.11 (0.05)	0.11 (0.05)
Male	−0.39*** (0.12)	−0.40 (0.35)	−0.10 (0.12)	−0.41 (0.34)	−0.03 (0.10)	0.53 (0.29)
Single parent family	−0.03 (0.17)	−0.03 (0.17)	−0.41* (0.16)	−0.41* (0.16)	−0.31* (0.14)	−0.31* (0.14)
Free school meal	−0.33 (0.20)	−0.33 (0.20)	−0.08 (0.19)	−0.06 (0.19)	−0.39* (0.17)	−0.36* (0.17)
English as additional language	−0.25 (0.14)	−0.25 (0.14)	−0.26 (0.14)	−0.26* (0.14)	−0.30* (0.12)	−0.29* (0.12)
Externalizing problems	−0.02 (0.06)	−0.02 (0.06)	−0.19** (0.06)	−0.18** (0.06)	−0.13* (0.05)	−0.12* (0.05)
CU traits	−0.03* (0.01)	−0.02 (0.01)	−0.02** (0.01)	−0.01 (0.01)	−0.02** (0.01)	−0.01 (0.01)
CU traits x Male		0.00 (0.02)		−0.02 (0.02)		−0.03* (0.01)
Random effects						
Level 2 (class) variance	0.08 (0.06)	0.08 (0.06)	0.00 (0.00)	0.00 (0.00)	0.04 (0.03)	0.04 (0.03)
Level 1 (child) variance	1.34* (0.10)	1.34* (0.10)	1.29* (0.09)	1.28* (0.09)	0.95* (0.07)	0.94* (0.07)
ICCs	0.06	0.06	0.00	0.00	0.04	0.04
Loglikelihood	1212.90	1212.90	1186.23	1185.25	1079.81	1075.56

*CU* Callous-unemotional

* *p* < 0.05. ** *p* < 0.01. *** *p* < 0.001

#### English Grades

The ICU total score but not externalizing problems was a significant predictor in Model 1. Older students and male (compared to female) students had lower English grades. The other sociodemographic variables were not significant in Model 1. In Model 2, there were no significant interaction effects between the ICU total score and gender. In Models 1 and 2, 6% of the variation in English grade was due to class after ICU total score, externalising problems and other covariate adjustments. Therefore, our independent variables explained some of between-class differences.

#### Math Grades

In Model 1, both externalizing problems and the ICU total score significantly predicted lower Math grades. Age and living in a single-parent family were also related to lower Math grades. The other sociodemographic variables were not significant and did not predict Math grades. In Model 2, there were no significant interaction effects between the ICU total score and gender.

#### Science Grades

In Model 1, the ICU total score and externalizing problems significantly predicted lower Science grades. Living in a single-parent family, free school meal eligibility and English as an additional language were related to lower Science grades. Age predicted higher Science grades, but gender was not a significant predictor. The CU traits by gender interaction was significant in Model 2. In Models 1 and 2, 4% of the variation in Science grade was due to class (as in the null model) even after adjusting for ICU total score, externalising problems and other covariates. Hence, our independent variables did not explain any of the between-class differences in Science grades.

**Fig. 1:**
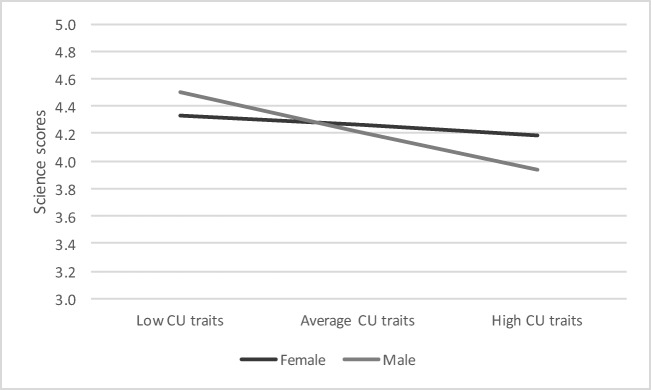
Predicted Science scores for children with low, average and high CU traits by gender. Notes. Low CU traits is defined as 1 SD below the mean (13.4), the average CU traits score is 21.28 and high CU traits is defined as 1 SD above the mean (29.16). Predicted values were plotted for individuals with mean values of continuous variables and for the reference group of the binary variables

To unpack the interaction effect, we plotted the predicted Science scores for illustrative cases of girls and boys with low (score one SD below the mean of 21.28 which equates to a score of 13.40), average (score of 21.28) and high levels of CU traits (score of 29.16, one SD above the mean) (Fig. 1). As is evident, the slope for boys is negative and shows that as CU traits increase, Science scores decrease. Although this negative slope appears for girls as well, it is flatter than for boys. The gap between boys with low CU traits (a Science score of about 3.93) and high CU traits (a Science score of about 4.50) is 0.57 points on the scale. For girls, this gap is only about 0.14 points on the scale.

## Discussion

Overall, results were consistent with our hypotheses as a direct link between CU traits and poor academic performance was evident for English, Science and Maths. Importantly, these associations were present even when controlling for both sociodemographic disadvantage and externalizing problems, well-established risk factors for poor cognitive and academic functioning in youth (Arnold 1997; Hinshaw 1992; Moffitt 1993). Past research has linked CU traits to poor academic achievement in Reading and Math achievement tests (DeLisi et al. 2011; Horan et al. 2016; Vaughn et al. 2011) and teacher ratings of overall academic performance (Ciucci et al. 2014; Fanti et al. 2017). Our study is the first to show that CU traits are also related to poor performance in Science as a separate discipline. The association between CU traits and low grades in all three disciplines indicates that this CU trait dimension is related to poor academic performance generally rather than being linked to a specific deficit in reading comprehension. Furthermore, the discipline of English includes not only reading but writing, grammar, vocabulary and speaking and thus English grades represent a broader index of academic performance than the reading comprehension tests used in previous studies (e.g., DeLisi et al. 2011; Vaughn et al. 2011). Current results fit with past research demonstrating that CU traits are related to poor outcomes in number of academic disciplines (e.g., Ciucci et al. 2014; Horan et al. 2016). However, the size of the effect of CU traits on academic performance was small, with coefficients ranging from −0.02 to −0.03 due to an increase in one point on the ICU scale.

Our hypothesis regarding gender was partly supported, with male gender significantly related to poor marks for English but not Math or Science. This is consistent with evidence showing that the female advantage is most evident for English during secondary school, with much smaller effects seen in Math and Science (Voyer and Voyer 2014). The superior performance of girls in English specifically may be due to stereotype threat, as boys are aware from a young age that parents and teachers expect girls to do better in English (Hartley and Sutton 2013). Thus boys may underperform in English due to low expectations and the low value afforded to this discipline for their gender, leading to low effort and motivation. There is also evidence showing that parents encourage girls to put more effort into this subject than boys, possibly due to parental perceptions that girls possess greater talent in English (Varner and Mandara 2014). These factors may have operated in isolation or combination to produce lower English grades for boys.

The finding that CU traits increased risk for boys in Science but not English or Math was unexpected, but may be attributable to the different learning format of Science compared to the other two disciplines. In secondary state schools in England, Science is the main curriculum area where pupils work together in groups, whereas Math, and to a lesser extent English, are more focussed on individual work (Baines et al. 2003). It may be that the greater severity of CU traits, externalizing problems, impairments in peer functioning and lower levels of empathy and social competence seen in boys compared to girls (e.g., Fanti et al. 2017; Haas et al. 2018; Stickle et al. 2012) reduces their ability to derive the well-documented benefits of positive peer relationships on academic engagement, motivation and achievement (Wentzel and Muencks 2016). Another possibility is that the emphasis on sequential, activity-based learning in Science means that students are unable to progress or complete classroom activities without receiving feedback and guidance from the teacher. Teachers may be less willing to provide this input to male than female students due to the aforementioned gender differences in CU traits and their likely impact on the quality of the teacher-student relationship. Finally, the current emphasis on promoting women in Science, Technology Engineering and Mathematics (STEM) may be viewed by girls high in CU traits as a means of attaining social status or dominance, and thus motivate them to engage more in Science learning than boys. Future research should include assessment of student characteristics as well as observation of peer and teacher-student interactions to determine whether these factors explain the significant interaction between male gender and CU traits in relation to Science but not the other two core subjects.

In the current study, older students achieved lower grades in English and Math. Key Stage 3 (years 7 to 9) is a stage of schooling in England known for a slow-down of pupil progress, attributed to a range of factors including poor support for socially disadvantaged students, lack of engaging and challenging teaching, and school leaders’ decision-making suggesting that this stage of schooling is ranked a lower priority than senior high school (Department for Education 2015). It may be that these risks accumulate across the middle school years, resulting in lower grades for older students in higher year groups for English and Math. Findings linking English as an additional language and variables indexing social disadvantage to lower Math and Science grades are consistent with past research (Department for Education 2015; Voyer and Voyer 2014). Finally, our results indicated that externalizing problems were related to low Math and Science, but not English grades. There is extensive evidence linking externalizing problems to poor school performance, with a range of both child dispositional characteristics (e.g., low verbal ability, impaired executive functions, temperamental risk) and environmental factors (poor quality schooling, conflictual teacher-child relationships) potentially contributing to this relationship (Moffitt 1993).

Current findings need to be interpreted in the context of study limitations. The present study sample consisted of non-referred children attending one secondary school, with lower rates of children from single-parent families and who have English as an additional language compared to national statistics. Therefore it is not clear how well our findings would generalize to samples that are more representative on these indices or to clinical or adjudicated samples. However, previous studies consisting of predominantly Hispanic/Latino and Black/African students from disadvantaged neighbourhoods also demonstrated a significant association between CU traits and poor academic performance (e.g., DeLisi et al. 2011; Horan et al. 2016; Vaughn et al. 2011). While our measure of externalizing problems showed good internal reliability and validity in our sample, it was derived from a measure of reward sensitivity rather than a formal measure of externalizing behaviours. The present research would be strengthened with the inclusion of parent and teacher report of CU traits and externalizing problems to obtain a more comprehensive view of child behaviour. Another limitation relates to the cross-sectional design of the study, prohibiting inferences about the direction of relationships. That is, it is not clear whether CU traits causes poor academic performance, or if poor school performance leads to increased CU traits, or if reciprocal effects are present. Given that all studies examining the relationship between CU traits and academic outcomes to date have been cross-sectional, longitudinal research should be conducted to establish whether this relationship persists over time. Furthermore, while this study provides evidence for associations between CU traits, gender and poor academic achievement, it does not shed light on the mechanisms behind these relationships. Explanations of the relationship between CU traits and poor academic performance focus on interactions between child characteristics (e.g., low academic motivation, punishment insensitivity, emotion processing deficits), and contextual factors, namely teacher-child interaction and teacher-student relationship quality (DeLisi et al. 2011; Horan et al. 2016). A multi-informant, multi-method longitudinal approach is needed to determine the nature and direction of these relationships and to identify mechanisms explaining the link between CU traits, gender and academic outcomes. In particular, classroom observation would be useful in providing an objective assessment of teacher-student interaction and teacher instructional methods.

Despite these limitations, the present study also features many strengths. This is the first study to examine the potential interaction between student gender and CU traits in relation to academic achievement. Findings therefore extend our understanding of how child characteristics may interact to increase impairment for youth high in CU traits in the under-researched academic domain. It is also the first study to examine links between CU traits and academic outcomes in the United Kingdom, with the bulk of past research conducted in samples largely comprising Hispanic/Latino and Black/African children attending schools in the United States. Research in different countries is important given differences in education policy and systems, teacher training, student assessment and other cultural influences. We considered it important to examine links between CU traits and academic performance in a mainstream secondary school given that previous research conducted during this period of schooling predominantly included students with known reading difficulties (DeLisi et al. 2011; Vaughn et al. 2011). Given that CU traits are not associated with deficits in verbal ability (Allen et al. 2013), their findings may not be representative of youth with CU traits. Another strength is the use of student grades based on curriculum set assessments across three major disciplines rather than teacher questionnaire ratings of school performance (Ciucci et al. 2014; Fanti et al. 2017) or standardized achievement tests (DeLisi et al. 2011; Horan et al. 2016; Vaughn et al. 2011). The academic advantage for girls is more apparent for teacher-assigned grades than for achievement tests (Voyer and Voyer 2014), while teacher ratings may be influenced by the student’s reputation, their disruptive behaviour in class or a poor quality teacher-student relationship, along with other well-known limitations of questionnaire report. Finally, we had a high student participation rate and accounted for the nesting of students within classes in our multilevel analyses.

Our findings suggest that boys high in CU traits are at increased risk for poor performance in Science. However, these findings are in need of replication as this is the first study to formally test the interaction between male gender and CU traits in relation to academic grades. Qualitative findings from teacher interviews suggest that poor academic achievement for children with elevated CU traits may be driven by a lack of motivation rather than a lack of ability (Allen et al. 2018). Therefore future research should assess motivation and other factors known to influence school engagement to better understand why such failures are occurring. School-based intervention has shown that targeting child (e.g., emotion understanding, social skills) and/or teacher factors (classroom management strategies) significantly reduces CU traits and externalizing problems (Frederickson et al. 2013; Kyranides et al. 2018). Our understanding of CU traits would be enhanced by research investigating whether these gains extend to academic progress following improvements in child behavioural and social adjustment.
